# Rethinking global health financing: from philanthropy to public good

**DOI:** 10.1186/s41256-025-00462-6

**Published:** 2025-12-09

**Authors:** Augustus Osborne

**Affiliations:** Institute for Development, Western Area, Freetown, Sierra Leone

**Keywords:** Global health financing, Philanthropy, Health systems, Equity, Public goods

## Abstract

Global health financing has been dominated by philanthropic and donor-driven vertical programs over the past two decades. While this model has achieved major public health gains, it has also entrenched power asymmetries, fostered fragmentation, and undermined the sustainability and sovereignty of health systems in low- and middle-income countries. This perspective critiques the ethical and practical shortcomings of donor-driven approaches, including the marginalization of local ownership and democratic accountability. The COVID-19 pandemic exposed the fragility of relying on voluntary, unpredictable funding and underscored the need for a shift toward treating health as a global public good. The paper argues for a transition to more sustainable models based on domestic resource mobilization, pooled multilateral funding, and participatory governance, and outlines concrete recommendations for reform. Transitioning to a public goods approach requires reforming funding mechanisms to enhance predictability and alignment with national priorities, embedding participatory budgeting and accountability frameworks, and investing in health systems and governance. A new social contract grounded in solidarity, equity, and shared responsibility is essential to secure health for all.

## Introduction

In the early twenty-first century, few sectors have been as profoundly shaped by philanthropy as global health. The Gates foundation, Gavi-the vaccine alliance (Gavi), the global fund to fight acquired immunodeficiency syndrome, tuberculosis and malaria, and a constellation of other private and public–private actors have revolutionized the landscape, channelling billions into the fight against infectious diseases, maternal mortality, and neglected tropical illnesses [1]. Their achievements - measured in lives saved, vaccines delivered, and new technologies deployed - are undeniable. Yet, beneath this apparent success lies a deeper, more troubling reality: global health financing has become increasingly dependent on a handful of wealthy donors and vertical, disease-specific programs, with priorities often set far from the communities most affected by ill health [1, 2].

For clarity, this paper defines key terms as follows: Philanthropy refers to large-scale funding from private foundations, such as the Gates foundation. Donor-driven approaches are those where priorities and strategies are primarily shaped by external funders (including philanthropic and bilateral donors) rather than recipient countries. Vertical programs are disease-specific initiatives with dedicated funding and delivery streams, such as those for polio or human immunodeficiency virus/acquired immunodeficiency syndrome. Finally, a global public good is a good or service whose benefits are non-excludable and non-rivalrous, extending across all nations, such as pandemic surveillance or climate stability.

This philanthropic turn, while filling critical gaps left by underfunded national systems and sluggish multilateral responses, may have fundamentally altered the balance of power in global health [3]. The big bets of billionaire philanthropists and international organizations now shape which diseases are targeted, which technologies are scaled, and which countries receive support [3]. As a result, the field is often driven by donor preferences rather than local needs or evidence-based priorities [3]. This model, celebrated for its agility and ambition, has been increasingly questioned for its sustainability, its ethical underpinnings, and its impact on health sovereignty and equity [4].

The COVID-19 pandemic has laid bare the fragilities of this system [5]. When global supply chains faltered and donor attention shifted, many countries found themselves scrambling for resources, exposing the dangers of dependency and the limitations of charity in the face of systemic threats [5]. As the world confronts a new era of complex, interconnected health challenges (climate change, antimicrobial resistance, and non-communicable diseases among them), the need to reassess the foundations of global health financing has never been more urgent. Should global health remain at the mercy of philanthropic largesse and fragmented vertical programs, or is it time to reimagine health as a global public good, sustained by collective public commitment and democratic accountability? The aim of this perspective is to critically examine the limitations of the current donor-driven and philanthropic approach to global health financing, assess the ethical and practical implications for health systems in low- and middle-income countries, and propose actionable reforms rooted in the concept of health as a global public good.

## The problems with donor-driven and vertical funding

The rise of philanthropic and donor-driven funding has been associated with a shift in how global health priorities are set [6]. Rather than being entrenched in the lived realities and expressed needs of communities, agendas are frequently determined by the strategic interests and personal convictions of donors, which are frequently headquartered in the Global North [6]. This has sometimes come at the expense of broader health system investments and the integration of care for other pressing health concerns, such as non-communicable diseases or mental health, which may not align with donor priorities [7].

Vertical programs targeting specific diseases with dedicated funding streams, supply chains, and monitoring systems have become the hallmark of this era [8]. While verticality has yielded dramatic gains in areas like polio eradication and childhood immunization, it has also fostered fragmentation and inefficiency because in many low- and middle-income countries, health workers are pulled between competing programs, each with its own reporting requirements and incentives, leading to duplication of effort and missed opportunities for holistic care [8].

Moreover, donor-driven agendas can distort national health strategies. Governments, eager to secure scarce external financing, may therefore reorient their policies and resources to align with donor priorities, even when these do not reflect the most urgent health needs of their populations [9]. This phenomenon, sometimes described as *mission drift*, undermines the sovereignty of recipient countries and perpetuates a cycle of dependency.

It is important to acknowledge that philanthropy and vertical programs have played indispensable roles in addressing acute public health emergencies and neglected diseases, particularly in settings where national capacity or political will have been lacking [8]. For example, vertical initiatives have led to dramatic reductions in polio and human immunodeficiency virus/acquired immunodeficiency syndrome incidence, and philanthropic funding has enabled rapid development and deployment of new vaccines and technologies. Furthermore, in some contexts, vertical programs have acted as catalysts for broader health system improvements [8]. An existing body of literature also points to the potential of the diagonal approach, which seeks to leverage disease-specific funding to strengthen health systems more broadly [6]. This approach attempts to balance the benefits of targeted interventions with the need for integrated and sustainable systems.

## Sustainability and dependency: the fragile foundations of charity

The second, and perhaps more insidious, problem with the current model is its lack of sustainability. Philanthropic funding, by its nature, is voluntary, time-limited, and subject to the shifting interests of donors and economic cycles. Unlike tax-based public financing, which is anchored in a social contract and political accountability, donor funds can evaporate overnight, leaving programs and populations in precarious limbo [10].

The volatility of donor support was starkly illustrated during the COVID-19 pandemic. As wealthy countries redirected resources to their own domestic responses, many global health initiatives suffered budget cuts and supply chain disruptions. The Global Polio Eradication Initiative, for example, faced significant setbacks [11], while efforts to tackle HIV, tuberculosis, and malaria lost momentum [12]. This dependency is not accidental but structural. Countries that rely heavily on external aid are unable to plan for the long term or build resilient health systems, leaving them ill-prepared to respond to new threats [13].

This fragility is further exacerbated by recent shocks to official development assistance (ODA) as several bilateral donors have cut their health ODA, and major multilateral initiatives face significant funding uncertainty [12]. For example, Gavi, the vaccine alliance, faces a multi-billion-dollar funding shortfall for its 2026–2030 period [13]. Such shocks may send tremors through the health systems of recipient nations, jeopardizing long-term programs and underscoring the profound risks of dependency on a volatile funding landscape.

## Ethical issues: power, ownership, and accountability

The ethical dilemmas at the heart of the current global health financing model revolve around power: who holds it, how it is exercised, and to whose benefit. The concentration of decision-making authority in the hands of a few major donors has created a profound asymmetry between those who fund global health and those who are meant to benefit from it [14]. Yet, this power is not subject to the same checks and balances that govern public institutions [14]. Philanthropic organizations are accountable primarily to their boards and founders, not to the served populations, as decisions about where and how to invest are often made with limited transparency or meaningful input from affected communities [15].

The erosion of local ownership is perhaps the most damaging consequence of donor-driven global health [16]. When priorities and strategies are set externally, national governments and local communities are relegated to the role of implementers, rather than architects, of their own health futures [16]. Externally imposed programs often fail to account for local context, culture, and capacity. Vaccine campaigns designed in Geneva or Seattle may overlook the logistical realities of rural clinics, leading to gaps in coverage and community mistrust [17]. The result is a persistent implementation gap, where well-funded programs achieve limited impact because they fail to resonate with local needs and priorities [18].

## Toward sustainable alternatives

If the current model is unsustainable and ethically fraught, what are the alternatives? The first is to strengthen domestic resource mobilization. Health financing entrenched in national budgets and progressive taxation is more predictable, accountable, and responsive to local needs than external aid [19]. A key benchmark in this effort is the 2001 Abuja declaration, in which African Union governments pledged to allocate at least 15% of their national budgets to health [20]. However, over two decades later, an analysis showed that only a handful of nations had met this target [21]. The failure to achieve this goal reflects the immense challenges many countries face, including restrictive fiscal space, high debt burdens, and competing development priorities, highlighting the need for both domestic political will and international financial reforms [20].

There are encouraging examples. Rwanda has made remarkable progress through a combination of community-based health insurance and government investment [22]. Thailand’s universal coverage scheme has achieved near-universal access through general taxation [23]. However, many low- and middle-income countries face significant fiscal constraints [24]. International financial institutions must therefore support countries in strengthening their fiscal capacity, including through debt relief and fairer global tax policies [24].

While domestic resource mobilization is crucial, it is not sufficient. Many health threats, like pandemics, antimicrobial resistance, climate change, are inherently transnational, requiring collective action and pooled resources at the global level. This is where the concept of health as a global public good becomes vital [25]. A global public goods approach calls for a shift from fragmented, voluntary charity to coordinated and mandatory contributions based on countries’ ability to pay [26]. Recent proposals, such as a global pandemic preparedness fund, reflect a growing recognition of this need [27]. Crucially, a public goods approach must be anchored in equity and justice, with inclusive and transparent decision-making [28].

## Recommendations for reform

To move beyond the limitations of philanthropy, global health financing must embrace reform that prioritizes predictability, coherence, and alignment with national priorities. A summary of these recommendations is provided in Table 1. One critical step is the transition from fragmented and project-based funding to pooled and multi-year financing mechanisms that empower countries to plan for the long term. A reformed financing model should also incentivize investments in health system strengthening such as workforce development and infrastructure rather than siloed and disease-specific interventions.

**Table 1:** Summary of key recommendations for global health financing reform

Recommendation	Key actions
Strengthen domestic resource mobilization	Invest in tax systems, support debt relief, align donor support with national strategies
Operationalize global public goods	Establish pooled, mandatory funding mechanisms with inclusive governance
Institutionalize participatory budgeting	Create formal structures for citizen and civil society engagement in health budgeting
Invest in local capacity	Support workforce development, data systems, and local manufacturing

Reforming financing is also a matter of governance. Participatory budgeting, which involves citizens and civil society in budget decisions, can help ensure that spending reflects real needs. Evidence from Uganda has shown that community engagement can improve service delivery and reduce corruption [29]. At the global level, multilateral agencies must also strengthen their accountability to member states and civil society through transparent reporting and formal channels for stakeholder input.

Ultimately, sustainable global health financing depends on the capacity of countries to manage resources, deliver services, and innovate solutions. Donor support should therefore focus on building local capacity in governance, research, and advocacy. This means investing in health worker training, leadership development, and supporting local manufacturing of vaccines and medicines to reduce dependency. Capacity-building is a long-term endeavour, requiring sustained commitment and a willingness to cede control to local actors.

## Conclusions

The dominance of philanthropic and donor-driven funding has delivered undeniable progress, but it has also entrenched power imbalances and fostered dependency. The COVID-19 pandemic, climate crises, and other global threats have exposed the fragility of a model built on voluntary charity. Looking forward, the imperative is to reimagine global health as a collective public good, grounded in solidarity, equity, and democratic accountability. This requires a new social contract built on several pillars: a commitment to sustainable, predictable financing; a radical shift toward participatory governance; and a relentless focus on building local capacity and resilient health systems. The path ahead will not be easy. It will require political courage and a willingness to challenge entrenched interests. But the stakes could not be higher. As the world has learned, health is not a commodity to be dispensed at the whim of the powerful, but a right that must be secured by all, for all. Only by embracing this vision can global health move from the age of philanthropy to an era of justice, partnership, and lasting progress. Reimagining global health financing as a collective public good requires ensuring sustainable financing, embedding participatory governance, and investing in local capacity. These reforms are essential to build a more equitable and resilient global health architecture. Future research should focus on evaluating the implementation and impact of these reforms across diverse contexts.

## Data Availability

Not applicable.
